# The Fer tyrosine kinase regulates an axon retraction response to Semaphorin 3A in dorsal root ganglion neurons

**DOI:** 10.1186/1471-213X-7-133

**Published:** 2007-11-30

**Authors:** Zoya Shapovalova, Kyrylo Tabunshchyk, Peter A Greer

**Affiliations:** 1 Queen's University Cancer Research Institute, Division of Cancer Biology and Genetics, Kingston, Ontario, K7L 3N6, Canada; 2 Department of Pathology and Molecular Medicine, Kingston, Ontario, Canada; 3 Department of Mechanical Engineering, University of Alberta and National Institute for Nanotechnology, NRC, Alberta, Canada; 4 Institute for Condensed Matter Physics, NASU, Lviv, 79011, Ukraine

## Abstract

**Background:**

Fps/Fes and Fer are the only two members of a distinct subclass of cytoplasmic protein tyrosine kinases. Fps/Fes was previously implicated in Semaphorin 3A (Sema3A)-induced growth cone collapse signaling in neurons from the dorsal root ganglion (DRG) through interaction with and phosphorylation of the Sema3A receptor component PlexinA1, and members of the collapsin response mediator protein (CRMP) family of microtubule regulators. However, the potential role of the closely related Fer kinase has not been examined.

**Results:**

Here we provide novel biochemical and genetic evidence that Fer plays a prominent role in microtubule regulation in DRG neurons in response to Sema3A. Although Fps/Fes and Fer were both expressed in neonatal brains and isolated DRGs, Fer was expressed at higher levels; and Fer, but not Fps/Fes kinase activity was detected *in vivo*. Fer also showed higher *in vitro *kinase activity toward tubulin, as an exogenous substrate; and this activity was higher when the kinases were isolated from perinatal relative to adult brain stages. CRMP2 was a substrate for both kinases *in vitro*, but both CRMP2 and PlexinA1 inhibited their autophosphorylation activities. Cultured mouse DRG neurons retracted their axons upon exposure to Sema3A, and this response was significantly diminished in Fer-deficient, but only slightly attenuated in Fps/Fes-deficient DRG neurons.

**Conclusion:**

Fps/Fes and Fer are both capable of phosphorylating tubulin and the microtubule regulator CRMP2 *in vitro*; and their *in vitro *kinase activities were both inhibited by CRMP2 or PlexinA1, suggesting a possible regulatory interaction. Furthermore, Fer plays a more prominent role than Fps/Fes in regulating the axon retraction response to Sema3A in DRG neurons. Therefore, Fps/Fes and Fer may play important roles in developmental or regenerative axon pathfinding through signaling from Sema3A to the microtubule cytoskeleton.

## Background

Fps/Fes (here after referred to as Fps) and Fer are the only two members of a subfamily of non-receptor protein tyrosine kinases. They are distinguished from all other tyrosine kinases by their unique N-terminal half, which consists of a Fps/Fes/Fer-Cdc42-interacting protein 4 (CIP4) homology (FCH) domain followed by an extended region of coiled-coils which collectively mediates their homotypic oligomerization [1,2]. The structures of the homologous domains in CIP4 and the related forming binding protein 17 (FBP17) [3] and FCH_0_2 [4] proteins display striking similarities to the membrane-binding BAR domains of Bin-Amphiphysin-Rvs family members; and it has been proposed to describe this domain as an F-BAR or extended FCH (EFC) domain [3].

Fps and Fer kinases also contain a central Src-homology 2 (SH2) domain, involved in binding to phosphotyrosine-containing peptide sequences [5]; and a highly conserved C-terminal kinase domain. The F-BAR domain of Fps and Fer kinases set them apart from all other tyrosine kinases. The conservation of these domains with a number of adaptor-like proteins involved in cytoskeletal functions, including FBP17, CIP4, PSTPIP1, PACSIN and cdc15p, as well as a subgroup of RhoGAPs, including the Slit-Robo GAPs, suggests that Fps and Fer kinases might regulate cytoskeletal and membrane reorganization associated with receptor endocytosis, secretion, vesicular trafficking, cell polarity and cell migration [6-9].

The role of Sema3A signaling in growth cone collapse has been extensively studied, however relatively little is known about how these signals lead to microtubule collapse and axon retraction. A complex of PlexinA1 and neuropilin 1 (NP1) was shown to act as a receptor for Sema3A [10,11]. More recent studies suggest that NP1 represses an PlexinA1/CRMP-mediated signaling pathway that leads to remodelling of both the actin and tubulin cytoskeletons [12-16]. Other studies have implicated PlexinA3 and A4 in Sema3A signaling [17,18] and Sema6C and 6D in PlexinA1 signaling [19]. Therefore, it is still unclear what degree of specificity or cross-reactivity exists between the numerous Plexin receptors and Sema ligands.

Fps was recently identified in a complex with microtubule-associated collapsin response mediator proteins (CRMPs and CRAM) in rat brain lysates [20]. In Cos-7 cell co-transfection studies, Fps promoted the phosphorylation of both CRMP/CRAM proteins and the Semaphorin receptor signal transducing subunit PlexinA1; and Sema3A promoted the PlexinA1 association with, and phosphorylation by Fps [20]. Transduction of DRG neurons with virus encoding kinase-dead Fps was also reported to inhibit Sema3A-induced growth cone collapse, but axon collapse was not investigated, nor was the potential involvement of the closely related Fer kinase in either process [20]. Subsequently these same authors showed that Fps was present at sites of microtubule nucleation in embryonic fibroblasts [21].

Purified Fps has been reported to catalyze the tyrosine phosphorylation of tubulin dimers *in vitro *and promote microtubule polymerization [22]. Fer has also been shown to localize at peripheral microtubules in polarizing and migrating vascular endothelial cells [23], and Fer has been implicated in regulating cortical actin dynamics downstream of cell-cell and cell-matrix receptor systems [24-31]. Together, these observations point to potential regulatory roles for Fps and Fer kinases in reorganization of both microfilaments and microtubules.

Here we show that Fer is more highly expressed and more active than Fps in the developing mouse nervous system. CRMP2 was a substrate for Fps and Fer; and both CRMP2 and PlexinA1 inhibited Fps and Fer activities *in vitro*. Using mice with targeted mutations in the *fps *and *fer *genes, we show that a Sema3A-induced axon collapse response was significantly compromised in Fer-deficient, but relatively normal in Fps-deficient DRG neurons. These novel observations provide genetic and biochemical evidence that the Fer kinase participates in signaling from Sema3A to microtubules, and might therefore play an important role in the microtubule collapse response associated with axon retraction.

## Results

### Fps and Fer are expressed in the developing nervous system and Fer displays evidence of *in vivo *kinase activity during neonatal brain development

**Figure 1:**
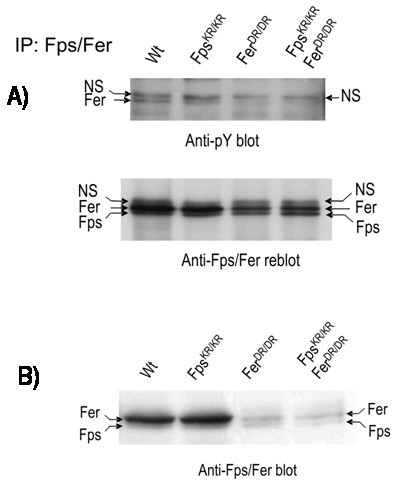
***In vivo *expression and tyrosine phosphorylation status of Fps and Fer in neonatal mouse brains and DRGs**. (A) Fps and Fer were immunoprecipitated from neonatal mouse brain lysates from the indicated mouse strains (*wt*, *fps*^*KR*/*KR*^, *fer*^*DR*/*DR *^or *fps*^*KR*/*KR*^*/fer*^*DR*/*DR*^) with anti-Fps/Fer antibody and immunoblotted with anti-pY (upper panel). The blot was stripped and reprobed with anti-Fps/Fer (lower panel). (B) DRG from indicated strains (*wt*, *fps*^*KR*/*KR*^, *fer*^*DR*/*DR *^or *fps*^*KR*/*KR*^*/fer*^*DR*/*DR*^) were dissected and subjected to immunoblotting with anti-Fps/Fer antibody. The positions of Fer, Fps and a non-specific (NS) band are indicated.

Fps and Fer were immunoprecipitated from neonatal brain lysates from: wild type mice (*wt*); mice targeted with a kinase-inactivating missense mutation in *fps *(*fps*^*KR*/*KR*^) [32]; mice targeted with a kinase-inactivating and protein-destabilizing mutation in *fer *(*fer*^*DR*/*DR*^) [24]; or compound mutant mice (*fps*^*KR*/*KR*^*/fer*^*DR*/*DR*^). Immunoprecipitation and immunoblotting were performed using an anti-Fps/Fer antibody that was previously shown to be equally cross-reactive toward these closely related kinases in both applications [33]. Immunoblotting with anti-phosphotyrosine antibodies revealed a specific signal at the expected migration position of Fer in *wt *and *fps*^*KR*/*KR *^mice (Fig. 1A, upper panel). This signal was diminished in *fer*^*DR*/*DR *^and compound mutant mice *fps*^*KR*/*KR*^*/fer*^*DR*/*DR*^, suggesting that it was tyrosine phosphorylated Fer. When these immunoblots were stripped and reprobed with the anti-Fps/Fes antibody, signals corresponding to both Fer and Fps were seen. However, the amount of Fer seen in *fer*^*DR*/*DR *^and compound mutant *fps*^*KR*/*KR*^*/fer*^*DR*/*DR *^mice was reduced (Fig. 1A, lower panel). This reduction in Fer levels was expected because the *fer*^*DR *^mutation causes a destabilization of the protein, in addition to abolishing its kinase activity [24]. Fps protein levels remained constant across all genotypes (Fig. 1A, lower panel) and there was no detectable anti-phosphotyrosine signal corresponding to the migration position of Fps in immunoprecipitates from any genotype (Fig. 1A, upper panel).

We next examined the expression of Fps and Fer specifically in DRGs dissected from neonatal mice (Fig. 1B). Immunoblotting analysis revealed that Fer was expressed at much higher levels than Fps in *wt *and *fps*^*KR*/*KR *^DRGs. In *fer*^*DR*/*DR *^and *fps*^*KR*/*KR*^*/fer*^*DR*/*DR *^DRGs, approximately equal levels of Fps (or FpsKR) and FerDR proteins were detected. This is consistent with our previous observations that the FerDR protein is unstable in comparison with *wt *Fer [24]. Collectively, these results show that Fer is expressed at substantially higher levels than Fps in whole neonatal mouse brain and DRGs; and furthermore, that Fer, but not Fps, is detectably tyrosine phosphorylated *in vivo*, and is therefore presumably active in neonatal brains.

### Fps and Fer kinases show highest levels of kinase activity in brain during perinatal stages of neuronal development; and there is more Fer than Fps activity

**Figure 2:**
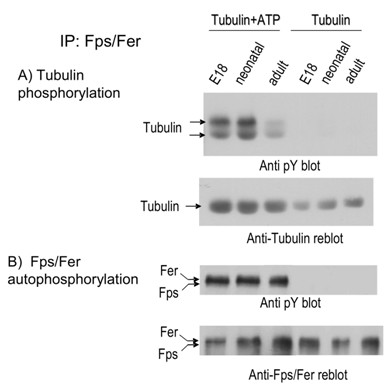
***In vitro *kinase activity of Fps and Fer isolated from embryonic, neonatal and adult mouse brains**. Whole brains from embryonic (E18), neonatal or adult *wt *mice were lysed in kinase lysis buffer and protein concentrations were determined. Fps and Fer were immunoprecipitated with anti Fps/Fer antibody from equal amounts of protein. The immune precipitates were processed for immune complex kinase assay with or without added ATP as described in Materials and Methods, and tubulin was added as exogenous substrate. (A), Supernatants containing tubulin were immunoblotted with anti-pY to reveal the degree of tubulin phosphorylation (upper). The blot was then stripped and reprobed with anti-tubulin antibody (lower). (B), Immunoprecipitated Fps and Fer proteins were dissociated from the beads and analyzed by immunoblotting with anti-pY to reveal the degree of *in vitro *Fps/Fer autophosphorylation (upper). The blot was then stripped and reprobed with anti-Fps/Fer antibody (lower).

**Figure 3:**
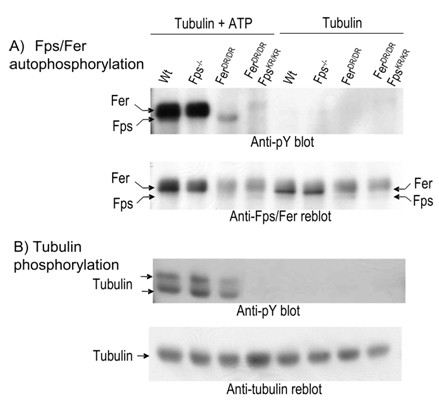
***In vitro *kinase activity of Fps and Fer isolated from neonatal mouse brains**. Fps and Fer were immunoprecipitated from neonatal mouse brain lysates from the indicated strains (*wt*, *fps*^-/-^, *fer*^*DR*/*DR *^or *fps*^*KR*/*KR*^*/fer*^*DR*/*DR*^) with anti-Fps/Fer antibody. The immune precipitates were processed for immune complex kinase assays with or without added ATP as described in Materials and Methods and tubulin was added as an exogenous substrate. (A), Beads containing immunoprecipitated Fps and Fer were immunoblotted with anti-pY to reveal the degree of Fps/Fer *in vitro *autophosphorylation (upper). The blot was stripped and reprobed with anti-Fps/Fer antibody (lower). (B), Supernatants containing tubulin were immunoblotted with anti-pY to reveal the degree of tubulin phosphorylation (upper). The blot was then stripped and reprobed with anti-tubulin antibody (lower).

We next examined the *in vitro *kinase activity of Fps and Fer at different stages of mouse brain development. Fps and Fer were immunoprecipitated from *wt *mouse brains of E18 embryos, neonates and adults. Immune complex kinase assays were then performed to assess the intrinsic autophosphorylation activity of these kinases, as well as their ability to phosphorylate the exogenously added substrate tubulin (Fig. 2). Comparable amounts of Fps plus Fer were present in these three stages of development (Fig. 2B, lower), and similar levels of *in vitro *kinase autophosphorylation were apparent (Fig. 2B, upper). However, the *in vitro *activity of Fps plus Fer toward tubulin was significantly higher in the neonatal and E18 stages than in the adult brain (Fig. 2A, lower). In these assays, the phosphorylation of tubulin might be due to the activity of Fps or Fer, or both kinases. Fps and Fer were not well resolved in these immunoprecipitates (Fig. 2B), relative to the lysates in Fig. 1. However, we reproducibly observed a fainter species migrating ahead of the major band in these immunoprecipitation experiments. This is consistent with a relatively greater amount of Fer to Fps, both in terms of mass amounts (Fig. 3B, lower) and autophosphorylation activity (Fig. 2B, upper). The identity of this species as Fps becomes clear in subsequent comparisons of *wt *and mutant samples (Fig. 3).

Gene targeted mice strains were next used to determine which kinase was responsible for the observed *in vitro *kinase activity in neonatal mouse brains. Brain tissues from *wt*, *fps*^-/- ^(targeted with a null mutation in *fps *[34]), *fer*^*DR*/*DR *^[24], or compound mutant *fps*^*KR*/*KR*^*/fer*^*DR*/*DR *^neonates [35] were subjected to immune complex kinases assays (Fig. 3). Compared to *wt*, the Fps plus Fer kinase autophosphorylation was only slightly reduced in *fps*^-/- ^lysates, substantially reduced in *fer*^*DR*/*DR*^, and abolished in *fps*^*KR*/*KR*^*/fer*^*DR*/*DR *^lysates (Fig. 3A, upper). From the migration positions and degree of tyrosine phosphorylation in the two bands we concluded that the faster migrating weakly phosphorylated band corresponds to Fps (see *fer*^*DR*/*DR *^lane in Fig. 3A, upper), while the slower migrating heavily phosphorylated band corresponds to Fer (see *fps*^-/- ^lane in Fig. 3A, upper). The apparent mass amounts of Fps and Fer in the anti-Fps/Fer immunoblots were also consistent with this interpretation (Fig. 3A, lower), and support the conclusion that there is more Fer than Fps in neonatal brains. *In vitro *phosphorylation of exogenously added tubulin was comparable in *wt *and *fps*^-/- ^lysates, substantially reduced in *fer*^*DR*/*DR*^, and abolished in *fps*^*KR*/*KR*^*/fer*^*DR*/*DR *^lysates (Fig. 3B, upper).

These assays were performed both in the presence and absence of ATP to control for the relatively low levels of *in vivo *tyrosine phosphorylation of Fps or Fer, which was not detectable under these conditions. These results indicate that Fer is the more active of these two related kinases in whole neonatal brain lysates; at least at the level of their apparent *in vitro *activity.

### PlexinA1 inhibits the autophosphorylation activities of Fps and Fer

**Figure 4:**
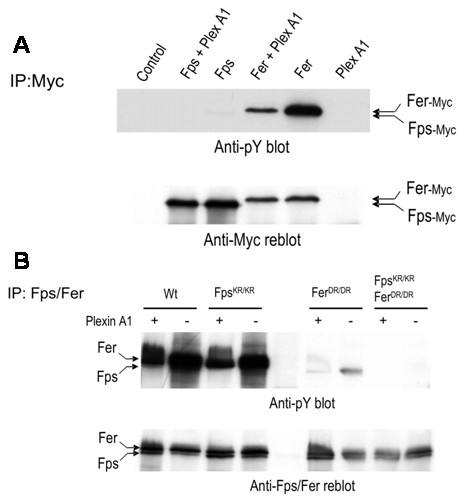
**PlexinA1 inhibits *in vivo *and *in vitro *Fps and Fer autophosphorylation**. (A), HEK293T cells were co-transfected with expression plasmids encoding Myc epitope-tagged Fps or Fer with or without PlexA1 as indicated. Fps or Fer were immunoprecipitated with anti-Myc antibody and immunoblotting with anti-pY was used to assess their degree of *in vivo *tyrosine phosphorylation (upper). The blot was then stripped and reprobed with anti-Myc to assess the level of Myc-tagged Fps and Fer expression (lower). (B), Fps and Fer were immunoprecipitated from neonatal mouse brains from animals of the indicated genotypes. Immune complex kinase assays were then performed with (+) or without (-) addition of purified GST-PlexA1-cyto (cytoplasmic domain) fusion protein as an exogenous substrate. Immunoblotting with anti-pY revealed the degree of Fps or Fer autophosphorylation (upper). The blot was then stripped and reprobed with anti-Fps/Fer (lower).

Fps was previously implicated in a Semaphorin signaling pathway in DRG neurons by binding to and phosphorylating both the Semaphorin receptor transducing subunit PlexinA1, and the downstream CRMP-CRAM complex [20]. Since we detected substantially more Fer than Fps expression in the developing brain and DRG (Fig. 1), we decided to investigate the relative involvement of Fer and Fps in the Semaphorin signaling pathway. PlexinA1 association with and phosphorylation by Fps or Fer were first examined in co-transfection studies. HEK293 cells were transfected with expression plasmids encoding Myc-epitope tagged Fps or Fer, with or without VSV-tagged PlexinA1. Anti-Myc immunoprecipitates were prepared from the cell lysates and immunoblotted with anti-pY (Fig. 4A, upper). No tyrosine phosphorylated bands corresponding to the size of PlexinA1 were observed in these Myc immunoprecipitates (data not shown), and no association of PlexinA1 with Fps or Fer was found in these co-immunoprecipitation analyses (data not shown). However, co-expression of PlexinA1 correlated with reduced autophosphorylation of both Fps and Fer (Fig. 4A, upper). In these *in vitro *assays, Fps was less active than Fer, but we could still see that Fps autophosphorylation activity was compromised by co-expression with PlexinA1.

A GST-fusion protein containing the complete PlexinA1 cytoplasmic domain was next tested as a purified exogenous substrate for Fps and Fer which was immunoprecipitated from neonatal mouse brains. A similar inhibition of both Fps and Fer autophosphorylation was observed *in vitro *when the purified GST-PlexinA1 protein was added as an exogenous substrate to anti-Fps/Fer immunoprecipitates from neonatal *wt*, *fps*^*KR*/*KR*^, *fer*^*DR*/*DR *^or *fps*^*KR*/*KR*^*/fer*^*DR*/*DR *^mouse brains (Fig. 4B, upper). There was no visible anti-pY signal corresponding to the migration position of the GST-PlexinA1 fusion protein in these assays (not shown), again suggesting that PlexinA1 was not acting as a substrate for Fps and Fer. These results argue that PlexinA1 can interact in some way with Fps and Fer kinases and modify their kinase activities, but not necessarily by acting as a substrate. Consistent with our previous results, Fer showed much higher intrinsic autophosphorylation activity than Fps, which can be seen by comparing the anti-pY signal in *wt *and *fps*^*KR*/*KR *^brain lysates with those from *fer*^*DR*/*DR *^or *fps*^*KR*/*KR*^*/fer*^*DR*/*DR *^mice (Fig. 4B, upper).

A "pull-down" assay using the GST-PlexinA1 cytoplasmic domain fusion was also performed to further test for possible association between PlexinA1 and Fps or Fer. HEK293T cells were transfected with Myc-epitope tagged Fps, FpsKR (kinase-dead), Fer or FerKR (kinase-dead) expression plasmids. Cell lysates from these cells were mixed with glutathione-agarose bound GST-PlexinA1 cytoplasmic domain or GST as a control. After washing, the specifically bound proteins were analyzed by immunoblotting with anti-Myc antibody. No association between PlexinA1 and Fps or Fer was detected using this method (data not shown).

### CRMP2 is a substrate for Fps and Fer, but inhibits their autophosphorylation activity

**Figure 5:**
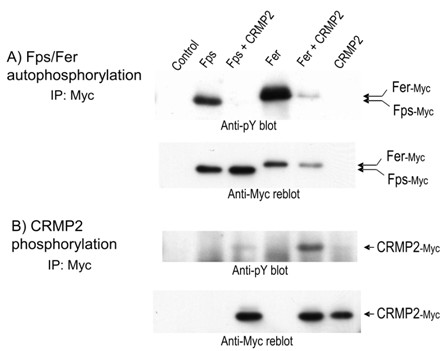
**CRMP2 is a substrate for Fps and Fer but inhibits their *in vivo *autophosphorylation activity**. HEK293T cells were co-transfected with expression plasmids encoding Myc-tagged Fps or Fer with or without Myc-tagged CRMP2. (A), The *in vivo *tyrosine phosphorylation status of Fps and Fer were assessed by immunoprecipitating them with anti-Myc antibody followed by immunoblotting with anti-pY antibody (upper). The blot was then stripped and reprobed with anti-Myc to reveal the level of Fps and Fer expression (lower). (B), The *in vivo *tyrosine phosphorylation status of CRMP2 was also assessed by anti-pY immunoblotting the anti-Myc immunoprecipitates (upper). The blot was then stripped and reprobed with anti-Myc to reveal the level of CRMP2 expression (lower).

Fps was previously identified in a complex with CRMP2 and CRAM in neonatal rat brains; and in co-transfection studies, Fps was shown to phosphorylate all four CRMPs [20]. We therefore investigated the possibility that Fer might also phosphorylate CRMP2. When HEK293T cells were co-transfected with Myc epitope-tagged CRMP2 together with Myc-epitope-tagged Fps or Fer, a band corresponding to tyrosine phosphorylated CRMP2 was observed in anti-Myc immunoprecipitates (Fig. 5B, upper). A comparison of the relative amounts of Fps and Fer (Fig. 5A, lower) with the degree of CRMP2 tyrosine phosphorylation suggests that CRMP2 was a much better substrate for Fer than it was for Fps under these conditions. Interestingly, the *in vivo *tyrosine phosphorylation status of both Fps and Fer were significantly reduced by co-expression with CRMP2 (Fig. 4A, upper). These results indicated that CRMP2 was a substrate for Fps and Fer. Furthermore, like PlexinA1, CRMP2 inhibited the *in vivo *autophosphorylation activity of Fps and Fer.

To test for associations between CRMP2 and Fps and Fer, this experiment was also performed using the anti-Fps/Fer antibody to isolate the Fps and Fer proteins, followed by immunoblotting with anti-Myc to assess the presence of associated CRMP2 protein. However, CRMP2 was not detected in these anti-Fps/Fer immunoprecipitates (data not shown).

### Fer regulates a Sema3A induced axon collapse response in DRG neurons

**Figure 6:**
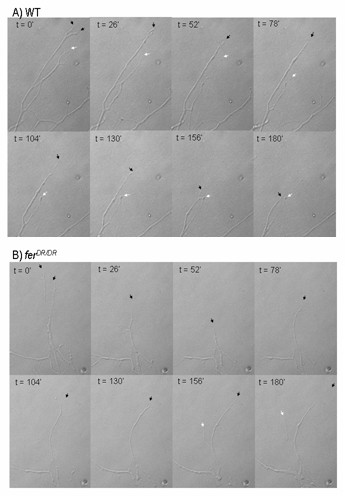
**Sema A-induced axon retraction**. Micrographs of representative images of data from Figure 7A at the time points indicated. The positions of axon ends are indicated with arrows. (A) *wt*, and (B) *fer*^*DR*/*DR*^.

**Figure 7:**
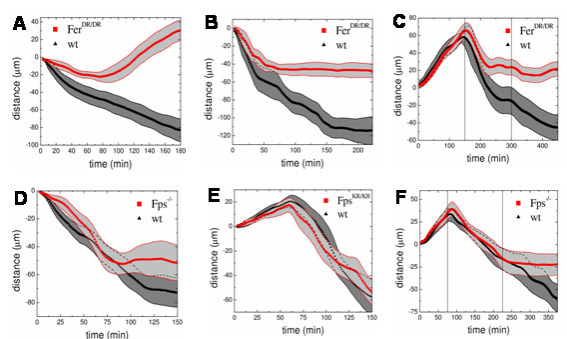
**Sema3A-induced axon retraction in *wt*, *fer*^*DR*/*DR*^, *fps*^-/- ^or *fps*^*KR*/*KR*^embryonic DRG neurons**. (A), Sema3A-induced *fer*^*DR*/*DR *^axon retraction in response to NGF. Sema3A was added to the NGF-containing media at time 0, and axon retraction quantified. X-axis indicates time of observation with the "zero" point corresponding to axon ends at time = 0. The Y-axis indicates the change in length of individual axons with decreasing or increasing distance values indicating axon retraction or growth, respectively. The black line indicates the mean value of *wt *axon behavior and the red line indicates the mean value of *fer*^*DR*/*DR *^axon response to Sema3A stimulation. Gray areas indicate standard deviations. (*wt*, n = 47 axons; *fer*^*DR*/*DR*^, n = 51 axons; *p *< 0.0005). Data was from 8 independent experiments using 12–18 embryos of each genotype. (B), Sema3A-induced *fer*^*DR*/*DR *^axon retraction in the absence of NGF. The analysis was performed essentially as in (A), except that before Sema3A stimulation, the NGF-containing media was removed and replaced with NGF-free media (*wt*, n = 35 axons; *fer*^*DR*/*DR*^, n = 39 axons; *p *< 0.001). Data was from 6 independent experiments using 8–12 embryos of each genotype. (C), *fer*^*DR*/*DR *^axon growth in the presence of NGF, retraction in response to NGF-depletion, and further retraction in response to Sema3A addition. The analysis was performed essentially as in A, except that axon outgrowth in the presence of NGF was monitored between 1–150 minutes (*p *< 0.3). The media was then replaced with NGF-free media and monitoring was continued between 150–300 minutes (*p *< 0.01). Finally, Sema3A was added and monitoring was continued between 300–450 minutes (*p *< 0.01) (*wt*, n = 29 axons; *fer*^*DR*/*DR*^, n = 27 axons). Data was from 5 independent experiments using 8–12 embryos for each genotype. (D), Sema3A-induced axon retraction of *fps*^-/- ^axons in the presence of NGF. This analysis was performed essentially as described in A, except using DRGs from *wt *and *fps*^-/- ^E14 embryos. (*wt*, n = 31 axons; *fps*^-/-^, n = 35 axons; *p *< 0.07). Data was from 4 independent experiments using 8–12 embryos per genotype. (E), Growth of *fps*^*KR*/*KR *^axons in the presence of NGF, followed by retraction in response to Sema3A. This experiment was done essentially as described above except axon outgrowth from *wt *and *fps*^*KR*/*KR *^DRGs in the presence of NGF was monitored from 0–60 minutes. Sema3A was then added to the NGF-containing media and axon retraction was monitored from 60–150 minutes (*wt*, n = 22 axons; *fps*^*KR*/*KR*^, n = 19 axons; *p *< 0.2). Data was from 2 independent experiments using 4–6 embryos for each genotype. (F), *fps*^-/- ^axon growth in the presence of NGF, followed by retraction in response to NGF-depletion, and further retraction in response to Sema3A addition. This experiment was done as described in C, except DRGs were from *wt *and *fps*^-/- ^E14 embryos. Axon outgrowth in the presence of NGF was monitored between 1–75 minutes (*p *< 0.4). The media was then replaced with NGF-free media and monitoring was continued between 75–225 minutes (*p *< 0.3). Finally, Sema3A was added and monitoring was continued between 225–375 minutes (*p *< 0.01) (*wt*, n = 36 axons; *fps*^-/-^, n = 32 axons). Data was from 4 independent experiments using 8–12 embryos for each genotype.

We next explored the potential involvement of Fer in regulating the response to Sema3A in DRG cultures. DRGs from *wt *and *fer*^*DR*/*DR *^E14 embryos were cultured overnight on collagen-matrigel in the presence of NGF to induce axon outgrowth. The cultures were then treated with Sema3A and their responses were documented by time-lapse video microscopy. Axons from *wt *embryos first retracted their growth cones (data not shown), and then gradually retracted their axons during the 3 hour observation period (Fig. 6A; Fig. 7; Additional file 1). Axons from *fer*^*DR*/*DR *^embryos also quickly retracted their growth cones upon Sema3A addition and also initiated an axon retraction response. However, the velocity of *fer*^*DR*/*DR *^axon retraction was slower over the first 80 minutes than was seen in *wt *axons. Perhaps more surprising, after 80 minutes *fer*^*DR*/*DR *^axons began to grow again in the presence of Sem3A (Fig. 6B; Fig. 7; Additional file 2).

The observed axon regrowth in *fer*^*DR*/*DR *^DRGs suggested that an NGF-induced outgrowth response might be competing with the Sema3A-induced retraction signal in the first 80 minutes, and that NGF response might completely overcome the Sema3A response after 80 minutes. This putative role of NGF was addressed in a second series of experiments. DRG cultures were again induced to grow axons overnight in the presence of NGF. The media containing NGF was then removed and replaced with the media containing Sema3A without NGF, and time-lapse video microscopy analysis was performed for 4 hours. The initial velocity of axon retraction was greater in *wt *than in the *fer*^*DR*/*DR *^DRGs, but the difference in the slope of this initial response was not as great as in the presence of NGF (compare Figs. 7A and 7B). By approximately 50 minutes, the rate of collapse of both *wt *and *fer*^*DR*/*DR *^axons had slowed. By 75 minutes the retraction response had terminated in *fer*^*DR*/*DR *^axons, but was still proceeding in *wt *axons. The late regrowth response that was initially seen when *fer*^*DR*/*DR *^axons were challenged with Sema3A in the continuing presence of NGF (Fig. 7A) was no longer observed when Sema3A was added in the absence of NGF (Fig. 7B). These results suggested that Fer promotes the Sema3A-induced axon retraction response, and might also inhibit the opposing NGF-induced regrowth response.

A third series of experiments were designed to further examine the involvement of Fer in the opposing NGF and Sema3A responses (Fig. 7C). DRGs from wild type and *fer*^*DR*/*DR *^embryos were again grown overnight in the presence of NGF to promote axon outgrowth. The kinetics of this NGF-induced growth phase was first assessed by time-lapse video microscopy for 150 minutes (Fig. 7C). Axon growth in the presence of NGF displayed a constant velocity with no difference between *wt *and *fer*^*DR*/*DR *^embryos (Fig. 7C). The culture media containing NGF was then removed and replaced with NGF-free media, and time-lapse analysis was carried out for another 150 minutes. Initially, axons from both genotypes collapsed at similar rates. However, *fer*^*DR*/*DR *^axons stopped collapsing approximately half-way through this NGF-depletion phase. In contrast, *wt *axons collapsed for a longer period of time, and only stabilized at the end of this NGF-depletion phase. Sema3A was then added and the analysis was continued for the final 150 minutes. *wt *axons displayed a characteristic Sema3A-induced retraction response which persisted throughout this final phase of the experiment. In contrast, *fer*^*DR*/*DR *^axons displayed no significant retraction response.

### Fps-deficient DRG neurons displayed only a subtle defect in the Sema3A-induced axon retraction response

Gene targeted knock-out (*fps*^-/-^) and kinase-inactive knock-in (*fps*^*KR*/*KR*^) mouse strains were next used to study the involvement of Fps in Sema3A signaling in mouse DRG cultures. DRGs from *wt *and *fps*^-/- ^E14 embryos were cultured overnight on collagen matrigel in the presence of NGF and then stimulated with Sema3A and subjected to time-lapse video microscopy (Fig. 7D). The analysis of mean values and standard deviations of *wt *and *fps*^-/- ^axon tracks did not reveal any statistically significant difference. However, the trend of *fps*^-/- ^axon retraction behavior was similar to that of *fer*^*DR*/*DR *^DRGs, suggesting that Fps might play a complementary role to Fer in the Sema3A response.

DRGs from *fps*^*KR*/*KR *^embryos were also analyzed for 60 minutes in the presence of NGF, followed by 90 minutes after addition of Sema3A in the continued presence of NGF (Fig. 7E). No significant differences were observed between *wt *and *fps*^*KR*/*KR *^DRGs in either the NGF-induced axon outgrowth or retraction upon Sema3A addition.

In a final set of experiments, *wt *and *fps*^-/- ^DRG cultures were first monitored in the presence of NGF for 75 minutes. Axon outgrowth rates were indistinguishable between *wt *and *fps*^-/- ^(Fig. 7F). The media was then changed to NGF-free culture media and monitored for 150 minutes. During this NGF-depleted culture phase, similar axon retraction rates were observed in *wt *and *fps*^-/- ^cultures. Sema3A was then added and the cultures were monitored for a further 150 minutes (Fig. 7F). *wt *axons continued to collapse after Sema3A addition, but *fps*^-/- ^axons appeared to stabilize during this final 150 minute period, showing no evidence of a retraction response to Sema3A. Previous studies have suggested that Fps might play a role in NGF signaling [36,37]. However, when DRG cultures from *wt *and *fps*^-/- ^embryos were allowed to extend axons in the presence of NGF, outgrowth rates were slightly higher in *fps*^-/- ^DRGs, however this difference did not reach statistical significance (Additional file 3). These observations, along with those in Fig. 7C argued that neither Fer nor Fps play major roles in NGF-induced axon outgrowth in DRG neurons.

## Discussion

A comparison of the *fps *and *fer *genes, and their encoded proteins, reveals a close evolutionary conservation, suggesting that they might perform homologous biological functions [6]. In a recent study by Mitsui and colleagues, Fps was identified in rat brain lysates in a complex with the microtubule regulating CRMP/CRAM complex [20]. Using transfection studies, they went on to show that Fps could interact with and phosphorylated the Sema3A receptor component PlexinA1; and that this stimulated the phosphorylation of CRMP/CRAM proteins. Virally transduced recombinant Fps could also regulate a growth cone collapse response to Sema3A in DRG neurons [20].

Given the close sequence similarity between Fps and Fer, we investigated the possibility that both kinases might be involved in the Sema3A signaling pathway to CRAM/CRMP; and subsequent microtubule regulation. We have shown here that both Fps and Fer are expressed in the developing mouse brain and in isolated DRGs. Fer was expressed to higher levels than Fps, and Fer had higher *in vitro *kinase activity toward the exogenous substrate tubulin. Fer was also detectably tyrosine phosphorylated *in vivo *in mouse brains, which suggested that it was actively participating in signaling events in the developing nervous system. Finally, we showed a statistically significant defect in Sema3A-induced axon retraction in DRGs from Fer-deficient mice (Figure 7A,B,C). A statistically significant defect in the Sema3A response of Fps-deficient DRGs was also detected, but only under conditions were NGF was depleted prior to addition of Sema3A (Figure 7F).

The study from Mitsui and colleagues used transfection studies to suggest a functional relationship between Fps and PlexinA1 [20], and the biochemical studies we report here also relied upon over-expression in transfected cells to suggest regulatory interactions between Fps and Fer with PlexinA1 and CRMP2. However, it should be noted that E14 DRG explants cultured in NGF will largely consist of PlexinA3- and PlexinA4-expressing nociceptors; so it seems unlikely that PlexinA1 would be involved in transmitting Sema3A signals to this type of neuron *in vivo *[18]. It would be interesting to test Sema3A responsiveness in Fps- and/or Fer-deficient DRG-derived proprioceptive neurons selected with NT3, which do express PlexinA1 [19]. Molecular analyses should also be extended to reconstituted Sema3A signaling pathways in cells transfected with different PlexinAs. This might reveal different specificities of Fps and Fer for interactions with the different PlexinAs.

In Cos-7 transfection studies, Mitsui and colleagues showed a Fps-mediated phosphorylation of PlexinA1 [20]. In similar transfection studies, we failed to observe PlexinA1 phosphorylation, induced by either Fps or Fer. However, consistent with Mitsui's report, we were able to demonstrate CRMP2 phosphorylation by Fps; but, we found that CRMP2 was a significantly better substrate for Fer than it was for Fps. Mitsui and coworkers also demonstrated that Fps co-immunoprecipitated with PlexinA1 in transfection studies [20]; however, we were unable to detect such an interaction, either in co-transfection studies, or using an *in vitro *pull-down assay with a purified GST-PlexinA1 fusion protein. Interestingly, Mitsui and coworkers did not detect an association between Fps and CRAM or CRMP in their Cos-7 transfection studies; but they did observe an anti-Fps immunoreactive protein in CRAM immunoprecipitates from rat brains [20]. Although we were unable to detect physical interactions between PlexinA1 nor CRMP2 with either kinase, we observed that both PlexinA1 and CRMP2 inhibited the autophosphorylation activities of Fps and Fer. A similar phenomenon has recently been described for plectin, a cytoskeletal linker protein which acts as a substrate and binding partner for Fer [38]. Given the apparent ability of PlexinA1 and CRMP2 to inhibit Fps and Fer autophosphorylation, we conclude that some interaction must have been occurring, but it failed to survive our immunoprecipitation protocol. In light of the recently solved crystal structure of the extended F-BAR domain from the adaptor-like proteins FBP17 and CIP4 [3], and FCH_0_2 [4]; and the demonstration that these F-BAR domains can mediate oligomerization, a more intriguing possibility is that substrates like CRMP2 and PlexinA1 might interact with the F-BAR domains of Fps/Fer kinases, and thereby compromise oligomerization or trans-interactions required for kinase auto-phosphorylation. The CRAM-Fps association observed in anti-CRAM immunoprecipitates from rat brain lysates by Mitsui and colleagues was presumably a very strong interaction to have survived the biochemical purification steps leading to Fps identification [20]. Since SH2-mediated associations with specific phosphotyrosine-containing peptides are typically high affinity interactions, it seems reasonable to speculate that such an interaction might have been involved in that association. However, while Mitsui and colleagues observed robust phosphorylation of CRAM and CRMPs when co-expressed in Cos-7 cells with Fps, they did not see co-immunoprecipitation [20]. It is possible that the N-terminal Flag epitope tags on these recombinant CRAM/CRMP proteins were permissive of phosphorylation, but sterically interfered with subsequent binding by the SH2 domains. The Myc epitope tag on the CRMP2 protein used in our study might have allowed phosphorylation, but could have interfered with subsequent SH2-mediated association. Similarly, it is possible that the VSV epitope tag on the recombinant PlexinA1 used in our study was permissive of associations that compromised kinase autophosphorylation, but precluded PlexinA1 phosphorylation, and subsequent high affinity associations with Fps or Fer. The PlexinA1 construct used by Mitsui and colleagues were C-terminally tagged with the HA epitope; and in Cos-7 cell experiments, this protein was both a substrate and a binding partner for Fps [20].

Among the most intriguing experiments performed in the Mitsui study were the reconstitutions of the Sema3A/NP1/PlexinA1/CRMP signaling pathway in transfected Cos-7 cells. Their observations are consistent with a model where NP1 restrains the cytoskeletal contraction signaling properties of a PlexinA1/Fps/CRMP pathway, while Sema3A relieves this NP1 inhibition [20]. Our studies in DRG cultures from Fps- and Fer-deficient mice are consistent with this model, but suggest that the Fer kinase might have an even more significant role in this pathway, relative to that played by Fps. In this regard, it is interesting to note that the two peptides originally identified in association with CRAM in rat brain lysates were compared to human and mouse Fps, with which they shared identical similarities (a collective 19 of 22 amino acids); but they were not compared to rat Fps and Fer. Interestingly, there is a slightly better conservation with rat Fer than rat Fps in these peptides (19/22 vs 18/22 amino acids). It will be important in future studies to compare the relative abilities of Fps and Fer to participate in this Cos-7 cell reconstituted Sema3A signaling pathway, and of course to evaluate the Sema3A collapse response in DRG cultures from compound Fps/Fer-deficient mice.

Kaibuchi and colleagues have shown that CRMP2 is involved in axonogenesis [39]. CRMP2 is highly enriched in growing axons, over-expression of wild type CRMP2 induced formation of multiple axons, and over-expression of C-terminally truncated CRMP2 inhibited axon formation [39]. CRMP2 binds to αβtubulin heterodimers and promotes microtubule assembly [40], and it also interacts with Numb to promote its role in endocytosis of the neuronal cell adhesion molecule L1 [41]. L1 is another component of the Sema3A receptor complex, and recycling of L1 is necessary for axon growth in DRG neurons [42]. CRMP2 is phosphorylated on threonine-555 by Rho kinase in response ephrinA5, and this blocked its tubulin binding and L1 endocytosis promoting properties, leading to growth cone collapse [43]. It will be important to map the Fps and Fer tyrosine phosphorylation sites in CRMP2 and determine if these phosphorylations affect its ability to bind to tubulin or microtubule regulator proteins.

Given the initial observations that Plexin proteins could be phosphorylated [11], it is interesting to note that several kinases in addition to Fps have been implicated in Sema3A signaling. These include Fyn, a member of the Src family of non-receptor tyrosine kinases; the Ser/Thr kinase glycogen synthase kinase (GSK)-3, and the Ser/Thr kinase cyclin-dependent kinase 5 (Cdk5). PlexinA1 and PlexinA2 were shown to associate directly with Fyn, and indirectly through Fyn with Cdk5. Fyn was also shown to phosphorylate Cdk5 and the PlexinA2 cytoplasmic domain [44]. Sema3A induced the phosphorylation and activation of Cdk5 by Fyn, which was essential for the collapse of sensory growth cones by Sema3A. Axons from explanted E17 Fyn^-/- ^DRGs showed an impaired response to Sema3A [44]. There is also evidence that Fyn might regulate Fps, and possibly Fer, as Fyn has been shown to phosphorylate a kinase-defective mutant form of Fps in Sf-9 cells [45], Fer and Fyn cooperate in actin depolymerization-induced cortactin phosphorylation [26], and the Fyn-related Lyn kinase regulates Fer phosphorylation in mast cells downstream of the IgE receptor engagement [46]. We have also found that Fyn is capable of phosphorylating kinase-inactive mutants of both Fps and Fer (data not shown). These observations suggest a potential regulatory relationship between Fps and Fer, and members of the Src family of kinases during Sema3A signaling. Since there are no obvious defects in the behaviour or activity of the Fer-, Fps- or compound Fer/Fps-mutant mice, we speculate that other kinases, perhaps members of the Src-subfamily like Fyn, might be able to compensate for loss of Fer/Fps kinases during development.

Fps has been shown to associate with microtubules by Takahashi and colleagues, who also observed Fps co-localizing with sites of microtubule nucleation and bundling in transfected Cos-7 cells; furthermore, the observed Fps co-localization with γ-tubulin at sites of microtubule nucleation was dependent upon the Fps FCH domain [21]. Purified Fps was also reported to phosphorylate tubulin and promote its polymerization *in vitro*; and biochemical association between Fps and soluble tubulin was dependent upon the Fps FCH domain [22]. Fer has also been shown to localize with peripheral microtubules in polarizing and migrating vascular endothelial cells [23]. In early stage hippocampal neurons, cell permeable peptide-based disruption of the Fer-p120catenin interaction correlated with a cell polarity defect, characterized by a delay in axon formation from a dominant neurite; and this phenotype correlated with deregulation of both the microtubule and actin cytoskeletons [8]. Interestingly, Fer has also been implicated in regulation of N-cadherin interactions in fibroblasts [25,31] and cross-talk from N-cadherin to integrins in neural retina cells extending neurites on laminin [47]; and in neuronal cell polarity and neurite development [8].

Both Fps and Fer were able to phosphorylate tubulin *in vitro*, and we found that the *in vitro *activity of Fps and Fer toward tubulin was highest in mouse brain tissues from perinatal stages, and significantly reduced in the adult. Thus, the highest levels of Fps and Fer kinase activity correlated with the developmental stage when a great deal of axon pathfinding activity is occurring. In contrast, Fps and Fer activities were greatly diminished in adults, when neuronal connections have been largely consolidated. *In vitro*, Fer possessed much higher levels of intrinsic autophosphorylation activity compared to Fps; and the ability of Fer to phosphorylate tubulin was also greater than that of Fps. Since tubulin is the major structural component of microtubules, the potential of Fer and Fps to phosphorylate tubulin *in vivo*, and the role that might play in microtubule dynamics will certainly merit further study.

Biological responses to Sema3A signaling are generally focused upon the growth cone collapse which occurs within minutes of ligand addition. In preliminary experiments using 0.25 μg/mL of recombinant Sema3A, we did see DRG growth cone collapse, but there was no significant differential response between *wt *and Fer- or Fps-deficient DRG neurons (data not shown). When these experiments were repeated with twice the Sema3A concentration, we again observed growth cone collapse with similar apparent kinetics; however, the axon retraction response in *wt *and *fer*^*DR*/*DR *^DRG neurons was significantly different. The *fer*^*DR*/*DR *^axons retracted with a slower velocity than those from *wt *DRG neurons, and at later time points *fer*^*DR*/*DR *^axons began to grow again, while *wt *axons continued to collapse. Our initial experiments were performed in the continuing presence of NGF, which presumably stimulated regrowth of *fer*^*DR*/*DR *^axons. Tuttle and O'Leary have reported that BDNF, NT-3 and NGF altered the sensitivity of growth cones to collapse in response to Sema3A, and NGF was shown to have the strongest protective effect against Sema3A-induced collapse [48]. Similarly, Dontchev *et al. *found that the Sema3A-induced collapse of growth cones of NGF-responsive embryonic sensory neurons was inhibited by NGF in a dose-dependent manner [49]. Apparently, these same protective effects of NGF extend to the axon retraction response. Furthermore, our observations indicated that in the absence of Fer kinase activity, NGF-induced axon regrowth is enhanced. This suggests that Fer might also play some role in signaling interactions between the NGF and Sema3A pathways.

Fer-deficient neurons were also defective in Sema3A-induced axon retraction in the absence of NGF. The removal of NGF might have also contributed to axon retraction. Therefore, in subsequent experiments we tried to distinguish between Sema3A-induced axon retraction and retraction caused by removing NGF. The result indicated that the removal of NGF promoted an axon retraction response in both Fer-deficient and *wt *neurons. However, the Fer-deficient axons stopped collapsing earlier than *wt *axons, and subsequent addition of Sema3A promoted further collapse of *wt *but not Fer-deficient axons. These results further supported the conclusion that Fer was required for the Sema3A-induced axon retraction. However, because the response to removal of NGF was slightly altered in Fer-deficient relative to *wt *DRGs, Fer might also be involved in other microtubule dynamic changes and reorganization other than that induced by Sema3A.

The removal of NGF from the DRG cultures might have induced a Wallerian-like axon degeneration response; and ultimately lead to apoptosis caused by loss of NGF-mediated survival signaling [50]. The initial kinetics of axon retraction seen upon NGF removal were similar in *wt *and *fer*^*DR*/*DR *^neurons (Figure 7C), suggesting that Fer was not involved in an early Wallerian degeneration-like response. However, we did note that *wt *axons continued to retract after *fer*^*DR*/*DR *^axons stabilized in the absence of NGF (Figure 7C). This suggests that Fer might be involved in signals contributing to axon retraction at these later times after NGF withdrawal. Since apoptotic signaling is also likely to be engaged by this time [51], future experiments should explore the potential role of Fer in neuronal cell death and survival signaling. This is a very interesting possibility, especially in light of recent work showing that Sema3A signaling contributes to apoptosis in NT3-, BDNF- and NGF-dependent embryonic DRG neurons [52].

*fps*^-/- ^DRG neurons displayed only a slight, yet statistically significant, defect in their Sema3A-induced axon retraction response; but unlike the *fer*^*DR*/*DR *^axons, no regrowth was observed in the presence of NGF. Axon retraction in response to removal of NGF was similar in *fps*^-/- ^and *wt *DRG cultures. However, the addition of Sema3A to the media promoted additional axon retraction of *wt *but not the *fps*^-/- ^axons. We therefore conclude that Fps is also involved in Sema3A signaling to microtubules. DRGs from Fps-kinase-inactive (*fps*^*KR*/*KR*^) mutant mice were also studied, but we found no difference in the Sema3A-induced axon retraction response, relative to *wt *axons. Since a slight defect was seen in Fps-null but not Fps-kinase-inactive cells, the data suggest a kinase-independent role for Fps in the axon retraction response to Sema3A.

Fps has previously been implicated in signaling downstream from the NGF in the PC12 cell system [36,37]. We studied axon outgrowth in response to NGF in DRG neurons from Fps- and Fer-deficient embryos, however, both Fps-deficient and Fer-defective neurons extended axons with similar kinetics to *wt *cells. This demonstrated that in DRG sensory neurons, Fps and Fer are not required for NGF-induced axon outgrowth.

## Conclusion

In summary, the work presented here provides the first evidence implicating the Fer tyrosine kinase in a Sema3A-induced axon retraction response signaling pathway. Although no obvious neurological defects have been observed in Fps-, Fer-, or compound Fps/Fer-deficient mice, this study provides strong justification for further work to elucidate the physiological importance of Fps and Fer kinases in the sensory nervous system and to investigate if compound Fps/Fer-deficient mice are compromised in their ability to sense their environments. More biochemical analysis is also required to determine if CRMP/CRAM proteins are substrates of Fps or Fer *in vivo*, how Sema3A signaling might regulate that phosphorylation, which PlexinA receptors participate in this Sema3A signaling *in vivo*, and how tyrosine phosphorylation of CRMP/CRAM proteins might affect their ability to regulate microtubule dynamics.

## Methods

### Expression constructs and transfections

Plasmids encoding Myc-tagged wild type Fer and Fps or kinase-inactive versions (FerKR and FpsKR) have been described previously [1,32,53]. Plasmids encoding Myc-tagged wild type CRMP2 and VSV-tagged Plexin A1 [54] were provided by Dr. Andreas Püschel. HEK293T cells were transfected using polyethylenimine as described previously [55]. Immunoprecipitations and immunoblotting were performed as described [1]. Blots were probed with the following antibodies:anti-phosphotyrosine (anti-pY) mouse monoclonal (PY99; 1:1,000; SantaCruz Biotechnology), anti-Myc mouse monoclonal 1-9E10 (1:10,000), anti-Fps/Fer rabbit polyclonal (1:1,000), anti-tubulin sheep pan-specific polyclonal (1:500, Cytoskeleton). Horseradish peroxidase (HRP) linked secondary antibody conjugates were whole sheep anti-mouse immunoglobulin G (IgG) (Amersham Biosciences) for anti-Myc and anti-pY antibodies, HRP-conjugated goat anti-rabbit IgG (Vector Laboratories) for anti-Fps/Fer antibody, or HRP-conjugated donkey anti-sheep IgG (Cytoskeleton) for anti-tubulin antibody in TBST for 1 h at RT. When reblotting with additional antibodies was required, blots were first stripped in 0.2 N NaOH twice for 15 min to remove the previous antibodies.

### Immune complex kinase assays

Brains were homogenized in 2 mL of KLB [1] using an Ultra-Turrax T25 homogenizer (Janke &Kunkel, IKA-Labortechnik). Lysates were clarified by centrifugation at 14,000 × *g *for 15min at 4°C. Protein concentrations were determined (Bio-Rad), and 1mg of extract was precleared with 25 μL of GammaBind-Sepharose (Amersham Pharmacia Biotech), then subjected to immunoprecipitation with either 5 μL of anti-Fps/Fer serum (equally reactive toward Fps and Fer) overnight at 4°C. Precipitates were washed three times in KLB and once in KRB (20mM Tris-HCl [pH 7.5], 10mM MnCl_2_, 100 μM sodium orthovanadate). Kinase reactions were performed by resuspending the immune complex in 30 μL of KRB supplemented with 1.5 μL of 10 mM ATP (Promega) and 0.5 μL of a 10 mg/ml stock of pure tubulin (minus glycerol) or 0.1 μg of purified GST fusion protein containing the cytoplasmic domain of PlexinA1. Kinase reactions were allowed to proceed for 20min at 30°C. After brief centrifugation supernatants containing tubulin were separated from the beads. Appropriate volumes of 6× or 2× SDS sample buffer were added to the supernatants or the beads, respectively. Samples were then heated for 5min at 100°C, resolved by SDS-PAGE and immunoblotting was preformed as described above.

### Transgenic mice

Generation and genotyping of in-bred SVJ/129-CD1 hybrid mouse lines bearing targeted catalytically inactive (*fps*^*KR*/*KR*^and *fer*^*DR*/*DR*^), and targeted null (*fps*^-/-^) mutations have been reported previously [24,32,34]. All mice were housed at the Animal Care Facility at Queen's University and all experimental procedures were approved by the University Animal Care Committee.

### Plasmid construction and isolation of GST-PlexinA1cyto fusion protein

Sequences encoding the cytoplasmic domain of PlexinA1 were PCR amplified from pBK-VSV-PlexinA1 using *Pfu *thermostable DNA polymerase (Stratagene) and 3' or 5' primers including EcoRI or BamHI restriction endonuclease cleavage sites, respectively (CCAGAATTCCGCTGCTCAGGGC or GTG GGATCCCTCATCGCCTACA), cloned between the *Eco*RI and *Bam*HI sites in the pGEX1-his plasmid and transformed into *Escherichia coli *DH5α. Soluble GST-PlexinA1-cyto protein was prepared as described [56].

### Embryonic DRG isolations, culture and Sema3A stimulation

Embryonic day 14 (E14) DRG were dissected from mouse embryos [57], and cultured on growth factor reduced BD Matrigel Matrix (BD Biosciences) in Delta T dishes (Bioptechs, USA) for 18–24 hrs in the presence of 50 ng/mL NGF 7S (Murine, Natural) (Invitrogen) to induce axon outgrowth. Delta T dishes with DRG cultures were placed on the warmed stage of a Meridian Insight Plus inverted microscope (Olympus Optical, Tokyo, Japan) and equilibrated for approximately 30 min. Control images were taken for 20 min or longer to record axon growth in the presence of NGF. Ten μL of pre-warmed media was added to the culture as a mock control and axon behavior was monitored for 20 min. Axon retraction assays were then performed by addition of 0.53 μg/mL Sema3A (recombinant human) (R&D Systems) and pictures were taken every 90 sec for 2.5 – 3hrs.

### Time-lapse video microscopy and data analysis

Time-lapse video images were acquired on a Meridian Insight Plus inverted microscope (Olympus Optical, Tokyo, Japan) equipped with Hoffman modulation contrast. Images were captured with a cooled CCD camera (Apogee Instruments Inc, Model KX85) every 90 seconds using an HMC 20× LWD 0.4NA objective, with Image Pro-Plus 5.0 software (Media Cybernetics). The microscope stage was fitted with a Bioptics Delta-TC3 temperature control system and heated lid (Bioptics, USA) with CO_2 _port to maintain routine incubation conditions at 37°C and 5% CO_2 _(Bioptechs, USA). The microscope was also equipped with a motorized XYZ stage (Ludl Electronics products LTD, NY, USA) controlled via Image Pro-Plus 5.0 software. This allowed for multiple areas on the same dish to be studied sequentially.

Images were exported as TIF files to Image Pro-Plus 5.0 software, converted to sequences for further analysis, and contrasted digitally in order to enhance discrimination of axons and growth cones. A tracking function was used to track manually the end of growth cones or axons. X and Y coordinates were measured for the ends of the individual growth cones or axons and exported to Microsoft Excel for data management. Programs for data analysis were written in Fortran language. Calculations were done for every axon where the distances between time points were added or subtracted for growth or retraction, respectively. The graphs were generated in Origin software. The mean values and standard deviations were also calculated using Origin program. The statistical significance of the results was assessed by calculating *p*-values. The *p*-values were obtained by independent group Welch's t-test, referring to the difference between *wt *and *fer *or *fps *deficient axons.

## Authors' contributions

ZS carried out the animal, cell and molecular biological and biochemical studies, and drafted the manuscript. KT wrote the software to analyze axon dynamics and performed the statistical analysis. PAG conceived of the study, and participated in its design and coordination and helped to draft the manuscript. All authors read and approved the final manuscript.

## Supplementary Material

Additional file 1 WTDRG. Video time-lapse analysis of *wt *DRG neurite collapse in response to Sema3A corresponding to representative data used to generate Figure 7A. Click here for file

Additional file 2 FerMutDRG. Video time-lapse analysis of *fer*^*DR*/*DR *^DRG neurite collapse in response to Sema3A corresponding to representative data used to generate Figure 7A. Click here for file

Additional file 3 Supplementary Figure. Responses of WT and *fps*^-/- ^embryonic DRG neurons to NGF Click here for file
