# Supplementary figures and images for: The Fer tyrosine kinase regulates an axon retraction response to Semaphorin 3A in dorsal root ganglion neurons
Source: BMC Dev Biol. 2007 Nov 30;7:133. doi: 10.1186/1471-213X-7-133 (PMC2217550; doi:10.1186/1471-213X-7-133)

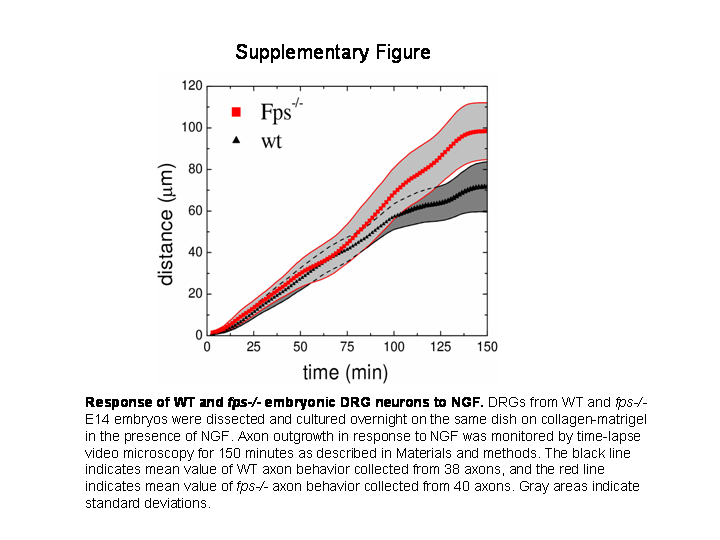

Supplement: Additional file 3 — Supplementary Figure. Responses of WT and fps-/- embryonic DRG neurons to NGF [file 1471-213X-7-133-S3.TIFF]
